# O.3.2-2 Substituting device-measured sedentary time with alternative 24-hour movement behaviours: compositional associations with adiposity, cardiometabolic risk and arterial stiffness in the ORISCAV-LUX 2 study

**DOI:** 10.1093/eurpub/ckad133.147

**Published:** 2023-09-11

**Authors:** Paul James Collings, Anne Backes, Gloria A Aguayo, Guy Fagherazzi, Laurent Malisoux

**Affiliations:** Luxembourg Institute of Health, Luxembourg; paul.collings@lih.lu; Luxembourg Institute of Health, Luxembourg; paul.collings@lih.lu; Luxembourg Institute of Health, Luxembourg; paul.collings@lih.lu; Luxembourg Institute of Health, Luxembourg; paul.collings@lih.lu; Luxembourg Institute of Health, Luxembourg; paul.collings@lih.lu

## Abstract

**Purpose:**

There is a considerable burden of sedentary time in European adults. We aimed to quantify the differences in adiposity, cardiometabolic risk biomarkers, and arterial stiffness associated with exchanging sedentary time for alternative 24-hour movement behaviours.

**Methods:**

This observational cross-sectional study included Luxembourg residents aged 18-79y who each provided ≥4 valid days of triaxial accelerometry (*n*=1046). Covariable adjusted compositional isotemporal substitution models were used to examine if replacing device-measured sedentary time with more time in the sleep period, light physical activity (PA), or moderate-to-vigorous PA (MVPA) was associated with waist circumference, numerous biomarkers of cardiometabolic health (fasting plasma glucose and insulin, triglycerides, high-density lipoprotein cholesterol (HDL-c), the ratio of apolipoprotein B/A1), and arterial stiffness (carotid-femoral pulse wave velocity (cfPWV)). We further investigated the cardiometabolic properties of replacing sedentary time accumulated in prolonged (≥30 minute) with non-prolonged (<30 minute) bouts. The results are presented as 30 minute time exchanges (β (95% confidence interval)).

**Results:**

Replacing 30 minutes of sedentary time with MVPA was favourably associated with waist circumference (-1.09 (-1.68 to -0.51) cm), HDL-c (1.30 (0.68 to 1.93) mg/dL), fasting glucose (-1.02 (-1.64 to -0.39) mg/dL), insulin (-4.71 (-7.12 to -2.24) %), and cfPWV (-1.70 (-2.76 to -0.62) %). Substituting sedentary time with light PA was associated with fasting insulin (-3.29 (-5.71 to -0.81) %), and was the only time-exchange to predict lower triglycerides (-2.32 (-4.38 to -0.21) %) and a lower apolipoprotein B/A1 ratio (-0.01 (-0.02 to -0.00)). Exchanging sedentary time with more time in the sleep period was associated with lower fasting insulin (-2.56 (-3.99 to -1.11)), and also with lower waist circumference (-1.39 (-2.30 to -0.48) cm) and cfPWV (-1.96 (-3.74 to -0.15) %) in short sleepers (<7h per night (*n*=349)). There was no evidence that replacing prolonged with non-prolonged sedentary time was related to outcomes.

**Conclusions:**

Replacing sedentary time with MVPA is beneficially associated with the widest range of cardiometabolic risk factors, but light PA confers additional and unique benefit. Extending sleep, by redistributing sedentary time, may be an appropriate strategy to lower obesity risk and improve arterial health in short-sleeping individuals.

**Funding:**

Luxembourg Institute of Health.

